# Increased 5-HT Levels Following Repeated Administration of Nigella sativa L. (Black Seed) Oil Produce Antidepressant Effects in Rats

**DOI:** 10.3797/scipharm.1304-19

**Published:** 2013-11-05

**Authors:** Tahira Perveen, Saida Haider, Nudrat Anwar Zuberi, Sadia Saleem, Sana Sadaf, Zehra Batool

**Affiliations:** 1 Neurochemistry and Biochemical Neuropharmacology Research Unit, Department of Biochemistry, University of Karachi, Karachi-75270, Pakistan.; 2 Department of Biochemistry, Jinnah Postgraduate Medical Centre, Karachi-75510, Pakistan.; 3 Department of Biochemistry, Jinnah University for Women, Karachi-74600, Pakistan.

**Keywords:** 5-HIAA, 5-HT, Antidepressant, *Nigella sativa* L., Tryptophan

## Abstract

The seeds of *Nigella sativa* L., commonly known as black seed or black cumin, and its extracts are used in folk medicine in the Middle East and in Asian countries for the promotion of good health and as a remedy for many ailments. These seeds have many acclaimed medicinal properties such as broncho-dilatory, immunopotentiating, analgesic, anti-inflammatory, and hypotensive. In the present study, the antidepressant activity following the repeated administration of *Nigella sativa* L. oil has been monitored using the forced swim test. Rats treated with *Nigella sativa* L. oil exhibited a significant increase in struggling time after oral administration of *Nigella sativa* L. oil (0.1 ml/kg/day) for four weeks. *Nigella sativa* L. oil increased brain 5-HT levels and decreased 5-HT turnover (5-HT/5-HIAA ratio). Levels of tryptophan increased significantly in the brain and plasma following the repeated administration of *Nigella sativa* L. oil. *Nigella sativa* L. oil showed a potential antidepressant-like effect.

## Introduction

Depression is among the most prevalent neurological disorders throughout the world [1]. It is mainly caused by the hypoactivity of neurotransmitters, particularly due to insufficient activity of serotonin (5-HT) [2]. Stress is the major precipitating factor in the onset of depression and this hypothesis is consistently supported by clinical observations [3]. Studies in experimental animals show that uncontrollable stress situations produce neurochemical alterations and behavioural deficits [3, 4]. These stress-induced behavioural deficits in animals are widely used as the animal model of depression. 5-HT plays an important role in the modulation of various behaviours. It is reported that a deficiency of 5-HT in the brain causes depression [5]. Increased 5-HT levels produce antidepressant effects. Administration of tryptophan, a precursor of 5-HT, has been shown to increase the concentration of brain 5-HT [6, 7] and produce antidepressant effects [8].

A large number of medicinal plants and their purified constituents have been shown to have beneficial therapeutic potential. Among the promising medicinal plants *Nigella sativa* L., a member of the Ranunculaceae family, is a useful herb with a rich historical and religious background [9]. *Nigella sativa* L. seeds contain fixed oil, volatile oil, proteins, alkaloids, coumarins, minerals, and carbohydrates [10]. It has been used for centuries both as an herb and oil in the Middle East, North Africa, and in Asia for the treatment of different diseases and also for general health. In view of the wide range of medicinal uses, this beneficial seed has underwent phytochemical studies for the isolation of various active compounds. Coumarin is one of the constituents of *Nigella sativa* L. and antidepressant activity of coumarin has previously been reported [11]. In addition, it has been shown that the seeds of *Nigella sativa* L. contain more than 30% of fixed oil and 0.40–0.45% w/w of volatile oil. *Nigella sativa* L. seeds and preparations thereof are frequently used as a natural remedy for many ailments such as headaches, nasal congestion, and toothaches [12]. Anti-inflammatory, analgesic [13, 14], antioxidant [15], and antihypertensive [16] activities of *Nigella sativa* L. has also been reported. Previous studies in our laboratory demonstrated that repeated administration of *Nigella sativa* L. oil enhances memory and produce analgesic effects [17]. Decreased 5-HT turnover and anxiolytic effects [18] of *Nigella sativa* L. have previously been reported. In the view of these findings, the present study is designed to investigate the antidepressant and neurochemical effects of *Nigella sativa* L. oil in rats.

## Materials and Methods

### Animals

Albino Wistar rats weighing 200–250 g, purchased from the HEJ Institute of Chemistry, University of Karachi, were caged individually under a 12:12 h light: dark cycle (light on at 6:00 h) and controlled room temperature (22±2°C) with cubes of standard rodent diet and water for 5 days before the experimentation. All experiments were performed according to a protocol approved by a local animal care ethical committee.

### Drug

The pure oil of *Nigella sativa* L. (black seeds) of Amir International purchased from a local market was used during the experiment.

### Experimental Design

Twelve rats were randomly assigned as the control and test groups. A dose of 0.1 ml/kg/day of *Nigella sativa* L. oil as reported previously [18] was given orally for 4 weeks to the animals of the test group. An equal volume of water was given to the animals of the control group. The forced swim test (FST) was performed following 4 weeks of drug administration to monitor the antidepressant effect.

### Behavioral Analysis

#### Forced Swim Test (FST)

The FST was used as a behavioural model to monitor the depressive symptoms in rats. The apparatus used in the present study consisted of a glass tank (53×19×28 cm) filled with water to a depth of 18 cm. At this depth, the rats were unable to support themselves by standing. The procedure and technique was essentially the same as reported by other researchers [19]. Rats were placed individually in the tank at room temperature for 5 min. After each test, rats were dried with a towel and placed back in their home cages. Behavioural scoring was performed by recording the struggling time. Struggling time was defined as an active behaviour which involved swimming, jumping, climbing the wall of the tank, and displacement around the surface of the water. The test is based on the idea that the water tank provides an inescapable stressed condition for the rats, comparable to the depressive situation in humans. Therefore, struggling time provides an index of antidepressive-like behavior as the rat tries to escape from the depressive situation.

### Neurochemical Analysis

At the end of the experiment, the animals were decapitated using a guillotine. The brain was removed immediately and stored at −70°C for the determination of TRP, 5-HT, and 5-HIAA by HPLC-EC as described earlier [20]. A 5-II Shim-Pack Octadecylsilane separation column of 4.0 mm internal diameter and 150 mm length was used. Separation was achieved by a mobile phase containing methanol (14%), octyl sodium sulfate (0.023%) and ethylenediaminetetraacetic acid (0.0035%) in a 0.1 M phosphate buffer at pH 2.9 on the Schimadzu Electro Chemical 6A Detector at an operating potential of 0.8 volts for biogenic amines and 1.0 volts for TRP.

### Statistical Analysis

The statistical analysis of the results was performed by using Student’s *t*-test. Results are presented as mean ± SD, with p values of > 0.05 considered as non-significant.

## Results

### Effect of Repeated Administration of Nigella sativa L. Oil on FST Activity

**Fig. 1:**
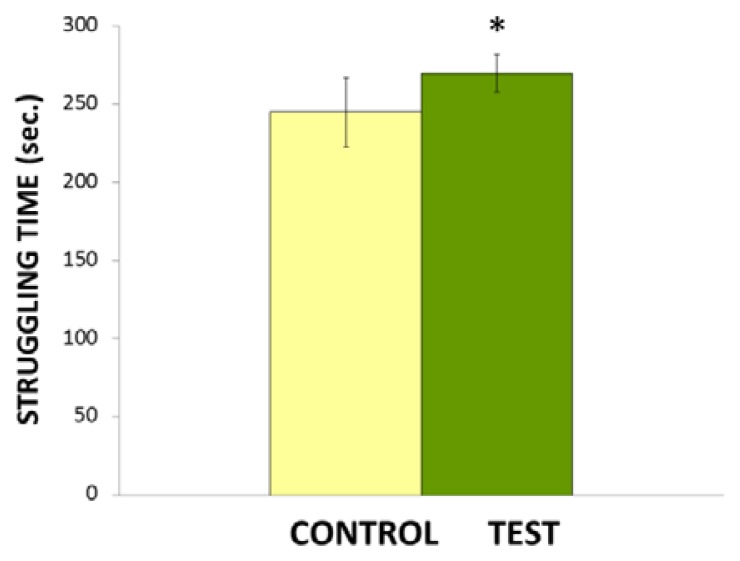
Effect of *Nigella sativa* oil on struggling time in a swim tank. Values are mean ± S.D (n=6). Significant difference by Student’s *t*-test *p<0.05.

Fig 1 shows the antidepressant-like effects of repeated oral administration of *Nigella sativa* L. in FST in rats. Statistical analysis by Student’s *t*-test reveals that repeated administration of *Nigella sativa* L. at the dose of 0.1 ml/kg/day significantly (*p<0.05) increased struggling time. This observed fact is taken to be an antidepressant-like effect of *Nigella sativa* L. and can be explained in terms of increased struggling time, where helpless despair syndrome is the minimum in test groups when exposed to FST.

### Estimation of Brain 5-HT Levels

**Fig. 2:**
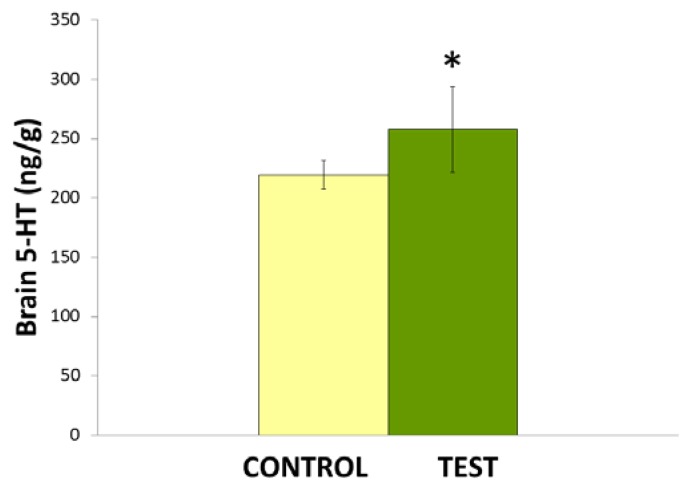
Effects of *Nigella sativa* L. oil on brain 5-HT levels. Values are mean ± S.D (n=6). Significant difference by Student’s *t*-test *p<0.05.

The effects of repeated administration of *Nigella sativa* L. oil (0.1 ml/kg/day) on brain 5-HT levels is shown in Fig 2. Statistical analysis by Student’s *t*-test shows that *Nigella sativa* L. oil significantly increased (*p<0.05) the 5-HT levels in rat brains.

### Estimation of 5-HT Turnover (5-HIAA/5-HT Ratio)

**Fig. 3:**
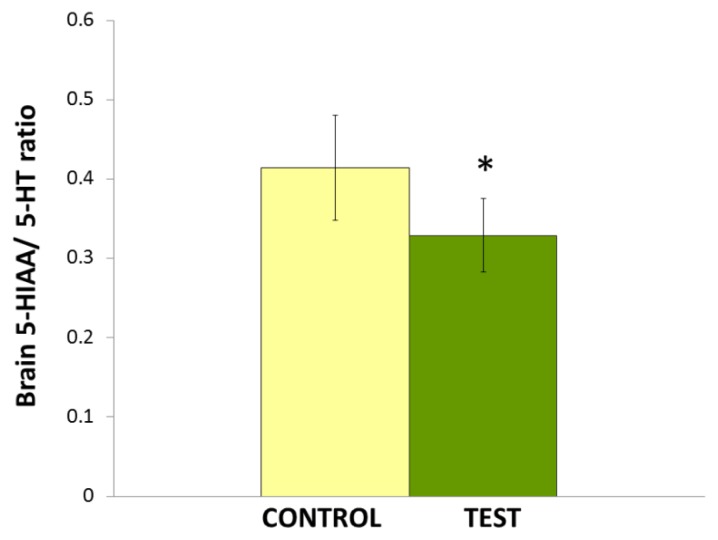
Effects of *Nigella sativa* L. oil on 5-HT turnover (5-HT/5-HIAA ratio) in the brain. Values are mean ± S.D (n=6). Significant difference by Student’s *t*-test *p<0.05.

The effects of repeated administration of *Nigella sativa* L. oil (0.1 ml/kg/day) on the 5-HIAA/5-HT ratio in the brain is shown in Fig 3. Statistical analysis by Student’s *t*-test shows that *Nigella sativa* L. oil significantly reduced (*p<0.05) 5-HT turnover in rat brains.

### Estimation of Tryptophan

**Fig. 4:**
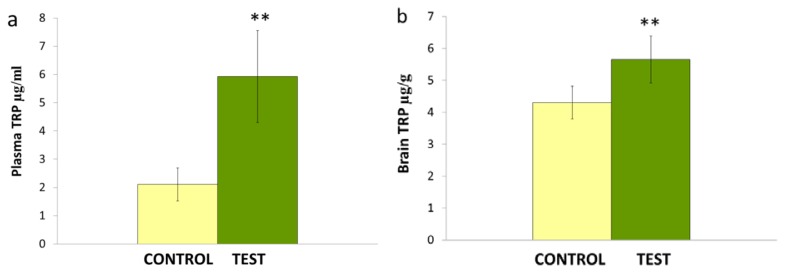
Effects of *Nigella sativa* L. oil on TRP levels in the plasma (a) and brain (b). Values are mean ± S.D (n=6). Significant difference by Student’s *t*-test **p<0.01 for plasma and brain TRP.

The effects of repeated administration of *Nigella sativa* L. oil (0.1 ml/kg/day) on tryptophan levels in the plasma and brain are shown in Fig 4. Data analysis by Student’s *t*-test shows that *Nigella sativa* L. oil significantly increased the plasma (**p<0.01) and brain (**p<0.01) tryptophan concentrations.

## Discussion

Aqueous [13] and phenolic [14] extracts obtained from *Nigella sativa* L. are used as a remedy for the treatment of various diseases, amongst them as a promoter of good health. *Nigella sativa* L. has been reported as an antioxidant [15], antihypertensive [16], anti-inflammatory, and analgesic [13, 14] as well as an anti-allergic agent [21].

The hypothesis that stress is a major factor in the onset of depression is consistently supported by clinical observations [22]. Uncontrollable stress situations produce neurochemical changes and behavioral deficits in experimental animals [3]. Stress-induced behavioral deficits in experimental animals are widely used as the animal model of depression. In the present study, the antidepressant activity of *Nigella sativa* L. was monitored by the forced swim test (FST). It was observed that *Nigella sativa* L. oil increased the struggling time in FST. The antidepressant activity of coumarin [23, 24] has also previously been reported. The role of life event stresses as a trigger of depression is known from many clinical surveys [25]. The classical hypothesis of serotonin’s (5-HT) function in depression describes 5-HT as an antidepressant compound, [26] and the deficiency of 5-HT is described as a proximate cause of depression [5]. Drugs that increase the 5-HT function are widely medicinally used as antidepressants [27]. The decreased turnover of serotonin indicated by a reduced steady-state concentration of the 5-HT metabolite, 5-hydroxyindoleacetic acid (5-HIAA), is a well-characterized effect of the centrally acting serotonergic agonist [28]. It has also been reported that the administration of monoamine oxidase inhibitors induces an increase in the levels of 5-HT and decreases the levels of its metabolite 5-HIAA [29] in rat brains. It was observed in the present study that repeated administration of *Nigella sativa* L. oil increased the levels of 5-HT and decreased 5-HT turnover in rat brains. This neurochemical profile and increased struggling time in FST, following the long-term administration of *Nigella sativa* L. suggests that *Nigella sativa* L. may act by potentiating the monoamine functions by inhibiting the activity of degradative enzymes. The inhibition of monoamine oxidase by coumarin derivatives has previously been reported [30].

It is well-known that the synthesis of serotonin in the brain depends upon the availability of its precursor, tryptophan, to the serotonergic neurons [31]. Increased brain tryptophan contributes to the enhancement of brain 5-HT metabolism [8, 32]. It has been reported that the repeated administration of *Nigella sativa* L. increases brain tryptophan [18] and 5-HT levels. Tryptophan hydroxylase, the rate-limiting enzyme of 5-HT biosynthesis, exists unsaturated with its substrate tryptophan [33]. Therefore, the factors that increase brain tryptophan also increase brain 5-HT synthesis [8]. The plasma levels of tryptophan have been shown to have a role in determining the brain tryptophan levels under different physiological and pharmacological conditions [34]. An increase in brain tryptophan concentration observed in the present study may be attributed to the increased concentration of plasma tryptophan levels.

## Conclusion

In conclusion, the present study suggests that the long-term administration of *Nigella sativa* L. oil may be highly useful in coping with stress as it increases the availability of 5-HT at synaptic sites by increasing plasma and brain tryptophan concentrations, thus increasing 5-HT synthesis and may also potentiate monoamine functions by inhibiting the activity of degradative enzymes leading to antidepressant-like effects. In the present study, the antidepressant-like effects of *Nigella sativa* L. oil observed by the increased struggling time in the forced swim test in rats suggests the promising use of *Nigella sativa* L. oil for the treatment of depression in humans.
